# Chronic Otorrhea—Its Significance and Treatment

**Published:** 1894-07

**Authors:** Dunbar Roy

**Affiliations:** Atlanta, Ga.; Professor Ophthalmology and Otology in Southern Medical College


### CHRONIC OTORRHEA—ITS SIGNIFICANCE AND TREATMENT.*

By DUNBAR ROY, A.B., M.D., ATLANTA, GA.,

Professor Ophthalmology and Otology in Southern Medical College.

My object in presenting this paper is not for the purpose of going into the intricate pathology of this morbid process, nor to bring forward any new and startling facts in its treatment, but in a practical manner to refresh the minds of those whose privilege it has not been to keep pace with the rapid advances in otology, and at the same time to incorporate some ideas gained from personal clinical experience.

*Read before the Georgia Medical Association, April, 1894.

Otorrhea etymologically means a discharge from the ear, but by common acceptation of the term it is usually understood as referring to a chronic running from external auditory meatus dependent upon a morbid state of the tympanic cavity. Every discharge from the ear has its source and its distinctive cause, which must be thoroughly ascertained before one can ever hope to treat successfully this class of troubles, for he who uses ear douches at random, or swabs out the ear in the same manner, will often come to grief, and such can never hope to rise to that prominence in his chosen profession which comes by exactness and knowledge of the subject, coupled with a large amount of common sense. In teaching at the Southern Medical College my habit has been to consider all chronic discharges from the ear as having their origin either in the external or middle ear.

1. CHRONIC DISCHARGES FROM THE EXTERNAL AUDITORY MEATUS.—(*a*) Discharges which usually occur in some portion of the human economy arise almost without exception from cavities lined by mucous membranes, and hence it looks quite anomalous that a discharge should arise from the external auditory canal which is nothing but a dermoid surface. That such is the case no one can doubt who has ever had much clinical work in ear diseases. Sir Joseph Toynbee, in his work on "Diseases of the Ear," published in 1860, enumerated many cases of pathological conditions of the external canal where one of its most common symptoms is a discharge from these parts. Sebaceous, sweat and ceruminous glands are abundantly scattered in the dermoid layer of the canal, and only need an irritative factor in any form to produce a hyperemia and congestion of the parts to be followed by a milky discharge. One of the most frequent causes of this symptom is a moist eczema of the canal, and closely allied to the same, as far as treatment is concerned, is the pathological condition known as otomycosis depending upon the presence of certain parasites for the production of this morbid state. The symptoms of the two conditions are much the same. On looking into the external auditory canal one will observe a wet, soggy condition of the parts, flakes of moistened epithelium like blotting paper clinging to the walls, and in addition, a turbid discharge exuding from the canal. Such a condition is not infrequently met with; hence it behooves the physician to be ever on the alert for the true diagnosis, since by the proper applications the parts can soon be restored to their normal condition.

The treatment consists in syringing the canal at the first sitting with a warm solution of biborate of soda, cleaning the canal with a small curette, drying thoroughly the parts with cotton and then applying your medicament. This latter is best done by ointments or powders. One of the best is a dram each of boric and salicylic acid to half an ounce of vaseline. This should be applied once a day, to be followed by insufflations of boric acid powder. With the subsidence of the symptoms the number of applications can be gradually decreased.

(*b*) *Polypi and Foreign Bodies.*—By the presence of such bodies as these the whole canal, through irritation, becomes congested, and with this hyperemic condition there is an increase of the glandular secretion in this part. The discharge is sometimes very copious, but differs from that which comes from the tympanic cavity in being more watery and whitish in appearance.

The only treatment in such cases as these is the removal of all the foreign and offending substances. Polypi are best removed with the cold wire snare and their base afterwards treated with chronic acid applications. If the growth is too small for the snare, then a small ring curette can be used.

2. DISCHARGES ORIGINATING FROM AND DEPENDENT UPON PATHOLOGICAL CONDITIONS OF THE MIDDLE EAR OR ITS CONTIGUOUS CAVITIES.—Let us refresh ourselves for a moment with the anatomy of the parts. The tympanic cavity is situated within the hardest bone of the body, communicating with the throat through the Eustachian tube, with the antrum mastoideus through the aditus ad antrum, while its outer wall is limited by the membrana tympani. It is lined throughout by mucous membrane, being continuous with that of the communicating cavities, and is traversed by a chain of three small ossicles. Two of these ossicles, the malleus and incus, are in close contact with the inner surface of the drum membrane, while the greater portion of their heads and bodies are situated high up in the attic of the tympanum. It is these two ossicles which have taken such a prominent part for the last few years in producing chronic discharges which had remained incurable until the necrosed ossicles had been entirely removed. Every case of chronic middle ear discharge can trace its origin to an acute otitis media, but why some of these acute cases recover without any untoward result, while others leave their mark in the shape of a chronic discharge, are questions which have not been satisfactorily answered. Certain it is that the condition of the general system, the question of blood and lymphatic dyscrasia play an important rôle. We must also bear in mind the fact that a chronic discharge from the tympanic cavity can be either *simple* or *purulent* in character, and in this way greatly influencing us in our prognosis. For convenience sake I have classified chronic discharges from the middle ear as occurring (1) childhood, (2) adults.

1. CHILDHOOD—*Causes.* (*a*) Children in whom there is a scrofulous or syphilitic taint are the ones most liable to chronic discharges from the ear, discharges from the nose, and to certain forms of ocular troubles, such as phlyctenular and interstitial keratitis. It is just in these cases that the systematic constitutional treatment produces its most beneficial results.

(*b*) The acute infectious and contagious diseases, especially scarlet fever and measles, are frequently followed by a chronic otorrhea as one of their sequela. In fact, statistics show that the majority of patients who suffer with a chronic discharge from the ear can trace its origin to an attack of measles or scarlet fever.

(*c*) Post-nasal adenoids, enlarged tonsils and chronic naso-pharyngeal catarrh are frequent causes and exciting agents in keeping up this condition through the connection of the middle ear and pharynx by means of the Eustachian tube.

2. ADULTS.—Most frequently can adult patients who suffer with chronic otorrhea from the tympanic cavity trace its origin to some period of their childhood. That the same factors which acted as causes in childhood may exist later in life is evident, but on account of the enlarging of the various parts and the greater power of resistance in the tissues of the adult, they are not so liable to the same disturbing influences. It is equally true that a certain amount of pathological conditions existing in the naso-pharynx in the way of adenoids, hypertrophies, chronic catarrh, etc., will not of themselves always answer as the cause for a chronic middle ear discharge, but we must look for some inherent condition existing in the middle ear itself as a cause for the persistency of the discharge. I have frequently seen all pathological conditions of the naso-pharynx relieved, and yet there would exist an occasional discharge from the ear. That there is a close relationship, however, existing between these parts all must admit, and he who ignores the nasal and naso-pharyngeal morbid states will never make a successful otologist.

When a chronic catarrhal condition of the middle ear has existed for some time, the mucous membrane and its contiguous parts are apt to be transformed into such a pathological state as to be themselves the existing causes of the same. When we remember that, with the exception of the outer wall, the middle ear is a cavity inclosed in hard bone where the nutrition of its lining mucous membrane is very easily interfered with, we are not surprised at the pathological conditions which we often find. Among the first such must be mentioned granulations upon the mucous membrane, which as long as they exist will be a source for the production of a chronic discharge. Almost without exception in a chronic middle ear discharge there exists a perforation of the drum membrane through which the discharge escapes; otherwise its only exit would be through the aditus ad antrum, or through the thin plate of bone which separates the tympanum from the brain, a result, not as Shakespeare says, "devoutly to be wished." Now, with this secretion in the tympanum, it matters but little how bland it is, one readily sees what a ready entrance is afforded to the various micrococci which will convert it into a purulent form.

Next in frequency must be mentioned polypi springing from the mucous membrane of the middle ear. These abnormal growths may be so small as to resemble granulations, while at other times so large as to protrude from the external auditory meatus. Most usually they are of the mucous type, but are by no means limited to one variety.

Finally comes a cause which of late has been recognized by otologists as playing such a prominent part in persistent purulent discharges from the tympanic cavity. This consists in a necrosis of some part of the bony walls of the tympanum, or of the chain of ossicles traversing the same.

*Treatment.*—Painstaking research into the history of every case; the correlation and comparison of all phenomena; a thorough knowledge of the normal and abnormal appearances of the parts, coupled with a sufficient amount of clinical experience—these are necessary for the successful treatment of all ear diseases. Though but an atom in size compared with the whole human frame, yet the field of otology is broadening every day, while the literature upon this one organ alone would yearly fill an ordinary case. Certainly there is no brighter outlook for the last three years in all the realm of medicine than is shown in the advances made in the surgery of the middle ear. While there may be extremists (but they exist everywhere), yet with the conservatism which is being manifested and the large amount of clinical experience which is being added daily to the literature of the subject, we may yet hope for more brilliant results. Every pathological condition must have its cause, and though we may not always be able to trace the same, yet it is our duty and own professional gain to go thoroughly into the history of every case, that we may determine, if possible, its *fons originis*. For instance, the large majority of children who suffer with a chronic otorrhea are also afflicted with some constitutional dyscrasia; hence it is only by the combination of the internal and topical applications that we can hope to bring about the most satisfactory results. It is not my purpose in this paper to treat of the internal medication, for that can properly be left to the good judgment of every physician, but rather to go more into the local management of these chronic discharges from the middle ear. Were I limited to one word as the foundation stone for the successful treatment of this class of diseases, it would be that of *cleanliness*. Every secretion which remains blocked up in any cavity is a continual source of irritation and the cause of other pathological changes, hence, for this reason, it should always be removed.

The treatment adhered to in children and adults differs but little, save that in the former the parts are more delicate, smaller and, in some degree, less accessible. Whenever the discharge is active and very profuse, especially in children, the secretion must be frequently removed by syringing the ear with some antiseptic solution. The best in my hands has been a saturated solution of boracic acid. In very young children have the parent or nurse use a soft rubber Davidson's ear syringe, and in this way the parts will not be injured should the child grow obstreperous. The physician himself should see the patient every other day, directing the nurse not to syringe the ear before coming to the office, in order that he may see any changes in the character and amount of the discharge. Having cleansed the ear out himself thoroughly by syringing, the physician should dry the parts with cotton. If now there exists a good sized perforation in the drum membrane, the middle ear should be thoroughly inspected. In young babies this is very difficult, so that frequently one has to be content with the following procedure, which so far has been eminently satisfactory: After syringing and drying the parts, the external canal is filled with pure listerine and allowed to remain for five minutes, when the parts are again thoroughly dried with cotton. The deep portion of the canal is now thoroughly insufflated with powdered boracic acid, the canal packed with iodoform gauze, its inner extremity having been dipped into iodo-carbolized glycerine, while its outer extremity is left accessible for removal by the nurse. The nurse is instructed to remove this packing as soon as it becomes saturated with the discharge, and again to commence using the frequent syringings with the boric acid solution.

This method of treatment is repeated every other day at first, and then at longer intervals. Having treated a number of chronic otorrheas in children and babies, by the procedure as above stated, I have found it by far the most successful in the large majority of cases. In adults the parts are more accessible, and likewise the pathological changes found more multiform. With this class of patients I find that the daily treatment, if possible, by the physician himself is much more successful, and likewise the time much shortened, than by allowing the patient to carry on the treatment at home, which, in the large majority of cases, is very unsatisfactory. If the perforation is large and in the lower half of the drum, the line of treatment as recommended in children, can be successfully used in these cases. Frequently, however, the perforation exists in the upper part of the drum and is very small in size. In such cases it is usually found that the discharge comes from the attic of the middle ear, and for the successful removal of the same, the solution must be introduced by means of a syringe or pipette of very slender and delicate proportions through the small opening in the drum. As a usual rule, when there has been a discharge for years, the whole or the greater part of the drum membrane is entirely destroyed. In such cases there exists usually some pathological condition of the mucous membrane of the middle ear, or some necrosis of the bony frame-work. Red, papillary granulations most frequently exist. These must be removed before you can hope to cure the discharge, as likewise polypi, which, as a rule, are only overgrowth of the granulations. The best way to remove the former is by curetting, and the latter either by the cold snare or the ring curette. With the removal of these pathological growths the middle ear must be continually treated with the antiseptic powders, combined with the tamponing of the auditory canal with iodo-carbolized gauze. If, as frequently happens, the patient is of such a neurotic temperament that instrumental manipulation in the ear is out of the question, then we may use what is termed the "alcohol treatment," instituted and highly recommended by Professor Politzer, of Vienna. This consists in the instillation of absolute alcohol into the middle ear, allowing it to remain for ten minutes at a time, to be repeated, at first, two or three times daily, and gradually diminished. At first the patient may not be able to endure more than a fifty per cent. solution, but this can be gradually increased until the full strength will be easily borne. I have seen most excellent results from this method of treatment, granulations disappear, polypi shrivel up and the whole ear reaches a healthy state.

Finally, comes a cause which of late has been found to play such a prominent part in keeping up a chronic discharge that has not yielded to the ordinary methods of treatment. This pathological condition is found to consist in a necrosis of some of the ossicles or bony walls of the middle ear, which, as long as it exists, will ever remain an exciting cause. Necrosis has been found most frequently in some portion of the malleus or incus, and since the greater part of these ossicles lie in the attic of the tympanum, one more readily suspects such a pathological condition when the discharge comes through a small perforation in the upper part of the drum. The diagnosis of necrotic bone is ascertained by means of the probe, and when such is discovered, there is but one course to pursue, that is the radical operation of thorough removal.

My paper has already reached a sufficient length to warrant me in not extending it further by going into the technique of the operation. At some later date I hope to publish my views upon the excision of the ossicles for both chronic otorrhea and for the relief of deafness.

56½ *Whitehall Street.*

---

SALICYLATES IN THE TREATMENT OF PLEURAL EFFUSIONS.

Dr. George Dock (*Therapeutic Gazette*) reviews the history of this subject, and draws the following conclusions:

1. Salicylic acid and its salts are among the most effectual agents in the treatment of pleurisy with effusion.

2. In effective doses the remedy is harmless, and with proper care in the selection of the preparation and its administration causes little or no discomfort to the patient.

3. Salicylates act most promptly in pleurisies with serous effusion of recent origin or of long standing, but they are efficient in simple dry pleurisy, and often act favorably in secondary pleurisy.

4. There is on evidence that they are useful in suppurative cases.

5. The drug acts as a diuretic, but may have an effect on the pathological process, or on the cause of the disease.

6. Salicylates have a more marked action on pleurisy than have the diuretics commonly so-called.

7. The duration of treatment with salicylic preparations is less than with diuretics, common salt, or roborant medication.

8. The remedy can be used at the earliest period, and favorably affects all symptoms.

9 The drug may be given in the form of the acid or any of its salts, in doses of a drachm of the former, or one to two drachms of a salt daily. In ordinary cases it is not necessary to give the large doses, and sixty to ninety grains of sodium salicylate or of salol may be considered full beginning doses, to be diminished one-third or one-half if the effect is manifest.

10 The ordinary precautions must be observed in giving the drugs, and during their administration the total amount of urine should be measured daily.—*University Medical Magazine.*

